# Breath-Holding as a Stimulus to Assess Peripheral Oxygenation Flow Using Near-Infrared Spectroscopic Imaging

**DOI:** 10.3390/bioengineering11121221

**Published:** 2024-12-03

**Authors:** Kevin Leiva, Isabella Gonzalez, Juan Murillo, Aliette Espinosa, Robert S. Kirsner, Anuradha Godavarty

**Affiliations:** 1 Optical Imaging Laboratory, Department of Biomedical Engineering, Florida International University, Miami, FL 33174, USA; kleiv004@fiu.edu (K.L.); igonz178@fiu.edu (I.G.); jmuri026@fiu.edu (J.M.); 2 Phillip Frost Department of Dermatology & Cutaneous Surgery, UM Wound Care Center, University of Miami, Miami, FL 33136, USA; a.espinosa2@med.miami.edu (A.E.); rkirsner@med.miami.edu (R.S.K.)

**Keywords:** near-infrared spectroscopic imaging, breath-holding, oxygenation flow, diabetic foot ulcers

## Abstract

A mammalian breath-hold (BH) mechanism can induce vasoconstriction in the limbs, altering blood flow and oxygenation flow changes in a wound site. Our objective was to utilize a BH paradigm as a stimulus to induce peripheral tissue oxygenation changes via studies on control and diabetic foot ulcer (DFU) subjects. Subjects were imaged under a breath-hold paradigm (including 20 s BH) using a non-contact spatio-temporal-based NIRS device. Oxygenated flow changes were similar between darker and lighter skin colors but differed between wound site and normal background tissues. Thus, the ability of peripheral vasculature to response to oxygenation demand can be assessed in DFUs.

## 1. Introduction

Approximately one-in-three people with diabetes mellitus (DM) will end up with diabetic foot ulcers (DFUs) during their lifetime [1]. If left untreated or poorly managed, DFUs can lead to amputations [2] and patients with DFU have an increased 5- and 10-year mortality. The current gold-standard clinical assessment of DFUs consists of visual inspection of the wound features (e.g., fissures, granulation, re-epithelialization), sensory feedback (warm to the touch, smell), and wound size measurements across weeks of treatment.

Oxygen is a vital component for wound healing [3]. In the presence of microvascular dysfunction, the wound healing process can be negatively impacted via restriction of the oxygenated blood flow to the site. Patients afflicted with cardiometabolic diseases, such as DM, are predisposed towards microvascular insufficiencies and may experience stagnated healing of their injured tissues. Transcutaneous Oximetry (TCOM) based devices are the gold-standard approach in assessing the wound healing status of DFUs [4]. TCOM is a non-invasive imaging approach that measures the partial pressure of oxygen diffused throughout the skin at discrete point locations around the wound site. That is, it measures the oxygen-delivering capacity of the vascular system.

TCOM devices utilize heating elements to induce tissue heating (~44 °C) as a stimulus to measure the partial pressure of oxygen under the skin. The dermal layer of the skin consists of a rich network of blood containing vessels [5] that are highly sensitive to thermal stress [6]. The upper papillary, layer of the dermis, is home to a large quantity of capillary loops used for thermal regulation. In response to increased body temperature, the capillary loops dilate to increase blood flow for improved heat transfer with the external environment [7]. Tissue heating caused by the TCOM device induces vasodilation in the capillary loops to better measure the amount of oxygen present in the assessed tissue.

TCOM is a time-consuming procedure (~30 min) and is performed as a contact-based imaging approach [8]. Due to the contact imaging nature of TCOM approach, it only provides information in the peri-wound at discrete point locations and not the entire wound bed and its surroundings. TCOM also requires additional equipment to increase the temperature around the wound, as a stimulus, to determine the partial pressures of oxygen. Thus, the applicability of TCOM as a bedside tool to assess all chronic DFUs during each visit or treatment is pragmatically challenging. There is a need for alternative non-invasive imaging techniques that can image the entire wound bed and its surroundings for these oxygenation changes to provide similar information to that of TCOM without the additional equipment and time-intensive procedures.

Various non-invasive optical imaging technologies have been developed to assess the tissue oxygenation distribution in and around the wound region. These include the non-contact hyperspectral imaging (HSI) [9,10,11,12], multi-spectral imaging (MSI) [8,13] and spatial frequency domain imaging (SFDI) [14,15] devices, apart from the contact-based near-infrared spectroscopy (NIRS) devices [16,17,18]. HSI and MSI obtain 2D maps of tissue oxygenation, via hemoglobin-based parameters, and compare them across weeks of treatment to assess the healing potential. While HSI and MSI are capable of measuring the effective amount of tissue oxygenation already present in the tissue, unlike the gold-standard TCOM, they are unable to assess how the vasculature responds to an oxygenation altering stimulus.

In our laboratory, we have developed a hand-held NIRS based imaging device, or a near-infrared optical scanner (NIROS). NIROS was developed for non-contact spatial-temporal tissue oxygenation monitoring as an indirect measure of perfusion. In this study, the imaging approach will utilize an innovative breath-hold (BH) paradigm as a stimulus to induce tissue oxygenation changes to the wound site [19]. Our hypothesis is that the mammalian breath-hold mechanism induces vasoconstriction in the limb, altering blood flow and tissue oxygenation. The characterization of breath-hold induced tissue oxygenation changes can be used as a stimulus to assess the ability of peripheral vasculature to respond to an oxygenation demand in diseased tissue models such as DFUs.

### 1.1. Breath-Hold Stimulus

All mammals possess an innate series of physiological mechanisms to conserve oxygen during periods of apnea. Breath-hold mechanisms prioritize brain oxygenation by triggering a series of cardiovascular changes to maintain cerebrovascular flow. Due to the simplicity in implementing breath-hold mechanisms, they have been used as a vasodilative stimulus in brain imaging [20]. These mechanisms require as little as 10 s of breath-holding to produce a Blood Oxygen Level Dependent (BOLD) signal [21]. Oxygen conservation is achieved through a combination of breath-hold induced bradycardia and peripheral vasoconstriction [22]. The extent of how much each physiological mechanism contributes to the conservation effect is affected by physiological factors (fitness, initial lung capacity), environment (wet/dry), and physical state (resting/exercising) of the participants.

Independent of any bradycardic response to breath-holding, vasoconstrictive mechanisms are engaged to reduce blood flow to the skin and muscles of the periphery [22]. It has been observed that skin blood flow could be reduced by 40% [23] and that muscle oxygenation decrease could be observed as early as 10 s [24] under breath-hold conditions. While the exact mechanism of the vasoconstrictive effect remains a topic of interest, it has been observed that the reduced blood flow to the limbs was due to reduced compliance in peripheral vasculature as opposed to a reduction in perfusion pressure [20].

### 1.2. Objective

In our previous pilot study [19], we implemented a 2 min breath-hold paradigm using (the previous version of) NIROS capable of only spatial imaging (not temporal) and obtained 2D tissue oxygenation maps at six discrete time points. The study was challenged with extensive motion artifacts during the 2 min imaging protocol. This was because the device used (a prior version of NIROS) could not dynamically map the changes in tissue oxygenation continuously. Hence, a longer breath-hold paradigm was utilized to have sufficient time-varying data points and map the tissue oxygenation flow synchrony or asynchrony. The study could not determine if the tissue oxygenation change occurred during or after breath-hold and what time-period of the entire breath-hold paradigm (total 2 min) was sufficient to capture the tissue oxygenation flow changes.

In this study, the NIROS device was modified to allow spatio-temporal mapping at 1 Hz frequency. This allowed continuous near real-time dynamic monitoring of tissue oxygenation changes in response to an external stimulus (e.g., breath-hold). Herein, the smallest time interval of the breath-hold paradigm that is sufficient to capture the tissue oxygenation flow changes from the vasoconstriction will be determined via studies on control subjects. The study will be systematically carried out on control subjects to: (1) validate that breath-hold does induce peripheral tissue oxygenation changes; and (2) determine the typical oxygenated flow patterns in control subjects on the dorsal and sole side of the foot using the chosen time-period within the breath-hold paradigm. We will also perform a preliminary assessment of the differences in breath-hold induced tissue oxygenation flow changes across control subjects, a healed DFU, healing DFUs, and non-healing DFU case with differing melanin concentrations.

## 2. Materials and Methods

### 2.1. Instrumentation

A continuous-wave, in-house built, near-infrared optical scanner (NIROS) was used to conduct non-contact imaging of the foot [19]. NIROS utilizes dual-wavelength (682 and 826 nm) near-infrared (NIR) LED packages. The NIR source light is diffused (via a diffuser sheet) prior to illuminating the tissue regions across a wide area. The diffusedly reflected NIR signals from the tissue passes through a 645 nm long pass filter (LP 645, MidOpt, Palatine, IL, USA) before being detected by a NIR sensitive CMOS camera (IDS, Obersulm, Germany). A custom LED driver is used to multiplex each wavelength at 1 Hz temporal frequency with 32 mW of optical power at the source end. The LED driver was optimized such that the source intensity and wavelength were stable during the dynamic imaging sessions up to 8 min. A Matlab-based graphical user interface (GUI) is used to automate spatio-temporal acquisition of diffusedly reflected signals from the NIROS device. Further details of the NIROS device are provided elsewhere [19].

### 2.2. Subject Recruitment and Experimental Paradigm

**Figure 1:**
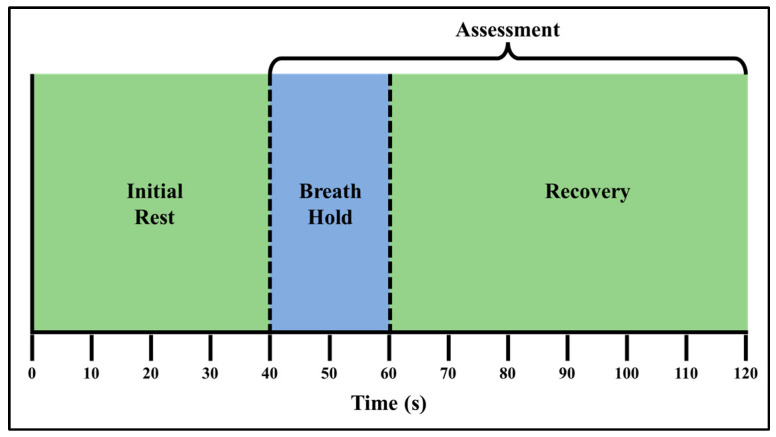
The 120 s long breath-hold paradigm, which includes three phases: initial rest (t = 0–40 s), end exhalation breath-holding (t = 41–60 s), and a recovery phase (t = 61–120 s). Tissue oxygenation changes were assessed during and after the breath-hold (recovery) phase.

Three control subjects were recruited via written consent on our approved IRB protocol (IRB-13-0092). The recruited subjects consisted of two females and one male subject, between the ages of 18–30 years and with a Fitzpatrick skin type of 1 [25]. The breath-hold stimulus can be performed as either a hold after inhaling air into the lungs (end-inhalation) or exhalation of air from the lungs (end-exhalation). In the current study, subjects were instructed to conduct end-exhalation breath-holds. The end-exhalation breath-hold paradigm was 120 s long and consisted of an initial rest, 20 s of end exhalation breath-holding and a recovery phase. An illustration of the breath-hold paradigm is given in Figure 1.

A preliminary study on 2 DFU cases was carried out to assess the effect of skin tone when imaging in response to the breath-hold paradigm. The details of the recruited DFU subjects are provided in Table 1 (Section 3.2).

### 2.3. Data Acquisition

Diffuse reflectance measurements in response to the breath-hold paradigm were acquired from each subject’s dorsal and sole of the left foot. As variation in the breath-hold induced tissue oxygenation changes may exist within subject, three repeated measures of breath-hold induced tissue oxygenated flow changes were acquired from both sides of the foot during a single visit. A 15 min rest period was provided across the repeated measures (3 repetitions) acquired from each side of each subject’s foot (dorsum and sole). Subjects were seated on a chair in the fowler position. Prior to imaging studies, the left foot of each subject was positioned onto a custom mount to minimize motion artifacts during the 120 s imaging study. Fiducial markers were placed distally on either side of the subject’s left foot for spatial referencing. Imaging was performed in a dark room with the device positioned ~15 cm away from the imaged foot. A uniformly diffusing calibration sheet (here, a disposable white cardstock) was used to capture the reference NIR signal across the imaged area (at both 682 and 826 nm wavelengths) prior to each imaging study [26,27].

### 2.4. Image Analysis

A custom in-house Matlab-based graphical user interface (GUI) was used to perform image analysis of the diffusely acquired NIR signals. The various steps in the image analysis process include: coregistering the time-varying diffusedly reflected NIR images to minimize motion artifacts, obtaining the spatio-temporal tissue oxygenation maps, extracting the time-varying hemoglobin concentration profiles, determining the oxygenation flow correlation maps, and calculating the oxygenation flow index (OFI).

Step 1: Coregistering the time-varying NIR images: Prior to evaluating the hemoglobin concentration maps from the dual wavelength diffusedly reflected NIR images, each image set was visually inspected for motion artifacts. The motion artifacts were corrected (or minimized) via an intensity-based coregistration algorithm. The coregistration technique automatically rotated and translated the NIR images to align with the NIR image acquired at the first time point during each imaging session. The geometric transformations of the NIR tissue images were accounted for in the reference images that were acquired using the calibration sheet.

Step 2: Spatio-temporal tissue oxygenation maps: The modified Beer–Lambert’s Law (mBLL) was utilized to calculate the spatio-temporal maps of the effective hemoglobin-based oxygenation using the coregistered diffusedly reflected NIR images of the tissue and calibration sheet [9,17]. The detailed analysis to obtain tissue oxygenation maps is described elsewhere [19].

Spatio-temporal tissue oxygenation maps were calculated in terms of effective oxy-(∆HbO), deoxy-(∆HbR), total hemoglobin (∆HbT), and oxygen saturation (∆StO_2_) for each case. These tissue oxygenation maps were calculated for each repetition study on the dorsum and sole sides of the left foot in each subject (excluding the toes).

**Figure 2:**
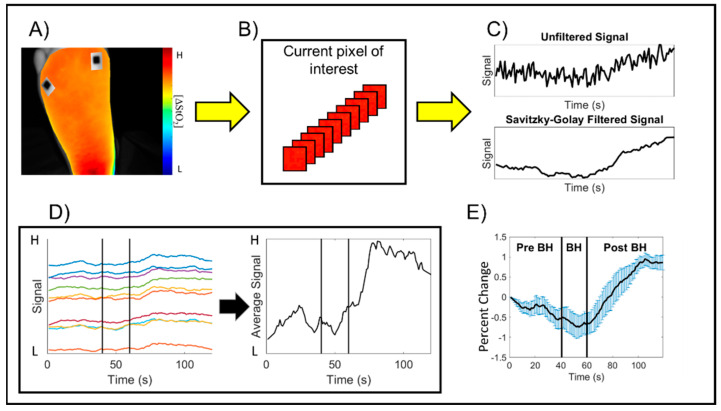
Schematic of the image analysis process to calculate the time-varying hemoglobin concentration profiles for an arbitrary subject and hemoglobin-based parameter. (**A**) A region of interest (ROI) encompassing the imaged foot tissue region was manually selected to remove the background region outside the foot. (**B**) The hemoglobin concentration changes at each pixel were extracted across time, and (**C**) The Savitzky–Golay filter was applied to each 2D pixel location across time to smoothen the signal while preserving the trend. (**D**) The average of the filtered signals was calculated to characterize the breath-hold induced hemoglobin concentration changes by subject. The average signal was initialized and normalized as the percent change with respect to breath-hold onset, where “H” represents High and “L” represents Low concentrations for the normalized hemoglobin-based parameter. Measurements from t = 41–120 s were further evaluated to assess the breath-hold induced hemoglobin changes. Each colored profile is a sample representation of the hemoglobin changes at various pixels within the selected ROI across time. (**E**) The grand average, or average of the average hemoglobin concentration signals, was used to characterize the breath-hold induced response for each subject, each imaged side of the foot, and each hemoglobin concentration parameter.

Step 3: Time-varying hemoglobin concentration profiles: In each spatio-temporal map, the region of interest (ROI) was defined as the imaged tissue region within the foot. The non-tissue background was segmented out from the spatio-temporal maps, and the changes in tissue oxygenation were assessed across the entire imaged region of the foot (as shown in one sample subject in Figure 2A). The time-varying effective hemoglobin-based concentration changes were extracted at each pixel location of the 2D spatial map. (Figure 2B). The Savitzky–Golay filter was applied at each pixel location across time to smoothen the signal while preserving the signal trend (Figure 2C) [28,29]. The time-varying hemoglobin concentration profiles acquired across each pixel was averaged across the entire ROI (Figure 2D). The averaged time-varying hemoglobin concentration profile from the onset time point of the breath-hold paradigm was considered for further analysis (i.e., t = 40 s onwards). This profile was initialized and normalized with respect to the first time point of breath-hold onset to obtain the percent change in tissue oxygenation signal. The average and standard error of the breath-hold induced hemoglobin concentration profile (from t = 40–120 s) was calculated from the grand average, or the average of the average, hemoglobin concentration signal across all 3 subjects and 3 repetitions for a given side of the imaged foot and parameter (Figure 2E).

Step 4: Oxygenation flow correlation maps: The oxygenated flow correlation maps were developed to assess synchrony or asynchrony in tissue oxygenation changes over the imaged region. The stimulus induced oxygenated flow was compared against a reference oxygenated flow signal across each 2D spatial pixel location that varied with time. The oxygenated flow correlation maps were generated using the time-varying ∆StO_2_ spatio-temporal hemoglobin concentration maps [19]. The oxygenated flow synchrony at each pixel location was calculated via linear correlation analysis to calculate the Pearson’s correlation coefficient (PCC). The PCC was calculated as given in Equation (1):

(1) $$PCC(x,y)=\frac{\sum_{i=1}^{n}\left(X-\bar{X})(Y_{i}-\bar{Y}\right)}{\sqrt{\sum_{i=1}^{n}{\left(X_{i}-\bar{X}\right)}^{2}{\left(Y_{i}-\bar{Y}\right)}^{2}}}$$

where $\bar{X}$ is the mean value of the reference signal across time, $X_{i}$ is the value of the reference signal at the *n*th time point, $\bar{Y}$ is the mean value of ∆StO_2_ of the *Y*th pixel across time, and $Y_{i}$ is the ∆StO_2_ value of the *Y*th at the *n*th time point. The output is a 2D Pearson’s correlation map that ranges from −1 (negatively correlated flow) to +1 (positively correlated flow). Each pixel location can be viewed as an individual correlation assessment of hemoglobin-based oxygenation changes at that pixel location against the reference signal. Oxygenated flow correlation maps were calculated for controlsubjects, and DFU cases in the current study. The average ∆StO_2_ across each 2D map was used as the reference signal for control subjects since they did not possess wounds. For DFU subjects, the ∆StO_2_ from a region of interest (ROI) in the background region (away from the wound site) was used as the reference signal. The ROI-based approach allows for direct comparison of background perfusion changes between the wound and peri-wound region.

Our preliminary analysis observed that distinct oxygenation flow changes were observed during the 20 s of breath-holding (t = 41–60 s), and within the first 20 s of the recovery, or post breath-hold phase (t = 61–80 s). Hence, flow correlation maps were generated using only this 40 s range of breath-hold induced changes. These findings are further expanded in the results (Section 3.3).

**Figure 3:**
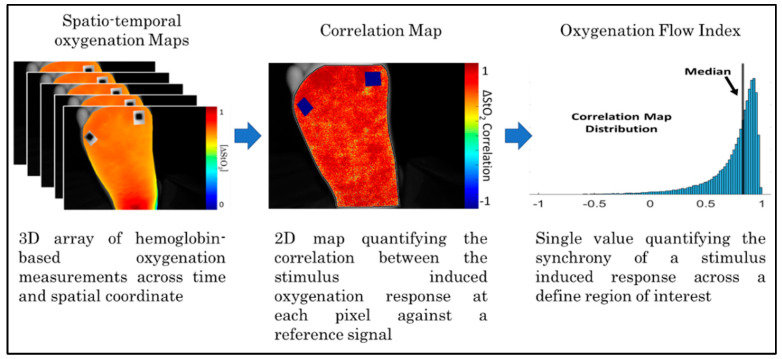
Summary block diagram detailing the steps required to calculate the Oxygenation Flow Index (OFI) values.

Step 5: Calculation of the Oxygenation Flow Index (OFI): The median value of the Pearson’s correlation coefficient distribution in each map was determined. The extracted median value of the correlation coefficient distribution was used as a measure to assess the overall extent of synchrony or asynchrony in each subject and the imaged side of the foot (dorsum or sole) (as shown in Figure 3). Using the median of the correlation coefficient distribution instead of the average of the distribution provides insight into the central tendency of the correlation distribution, even in potentially skewed distributions. Herein, the median value of the correlation coefficient distribution in each map is referred to as the oxygenation flow index (OFI).

## 3. Results

### 3.1. Time-Varying Hemoglobin Concentration Profiles of Control Subjects

**Figure 4:**
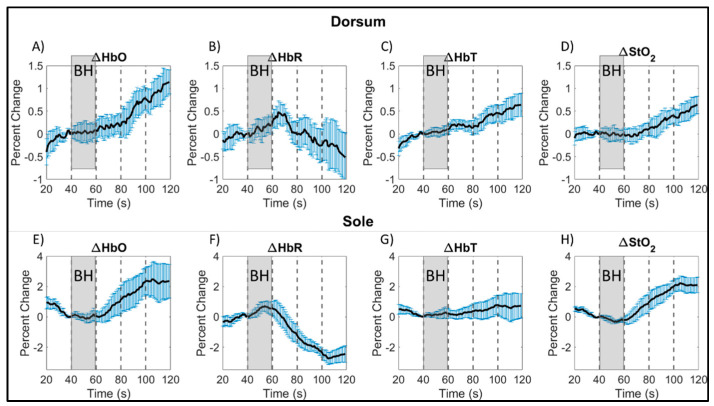
Average breath-hold induced hemoglobin concentration changes, calculated as the percent change from breath-hold onset, across all control subjects (and repetitions) by side of the foot for: oxy- (**A**,**E**), deoxy- (**B**,**F**), total hemoglobin (**C**,**G**), and oxygen saturation (**D**,**H**). Average signals were plotted with the standard error (blue vertical bars) calculated from all subjects at each time point. The gray shaded region denotes the breath-hold (BH) phase, and every 20 s of the post breath-hold (or recovery) phase is shown in increments as denoted by black, dashed, vertical lines. Note: The hemoglobin concentration profiles in the dorsum and sole of the foot (in terms of ∆HbO, ∆HbR, ∆HbT, and ∆StO_2_) could have changed even during the rest period (20–40 s) probably from an altered breathing pattern in preparation for their 20 s end-exhalation breath-hold.

The hemoglobin concentration profiles in terms of ∆HbO, ∆HbR, ∆HbT, and ∆StO_2_ across the 20 s of breath-holding (t = 41–60 s) and the 60 s of post breath-hold (t = 61–120 s) were calculated for each subject. The average profile for each of these hemoglobin concentrations were calculated across all control subjects and repetitions, for each side of the imaged left foot and given in Figure 4. An increase in ∆HbR was observed during the 20 s breath-hold phase. There was an increase in ∆HbO, ∆HbT, and ∆StO_2_ end of the 20 s breath-hold and in the post breath-hold (recovery) phase in the foot’s dorsum and sole. On the contrary, the ∆HbR dropped during the recovery phase. This trend of increased oxyhemoglobin [24] and oxygen saturation [30] post breath-hold is in sync with the literature’s report. Also, this increase in oxyhemoglobin and oxygen saturation was distinct within the first 20 s of the recovery period itself. Hence, the correlation maps were generated using only the 40 s timespan encompassing the 20 s recovery period along with the 20 s breath-hold period. This reduces the overall computation while still generating flow correlation maps that include the changes in oxygenation in response to the breath-hold stimulus. Additionally, the hemodynamic response to breath-hold was similar across the three control subjects’ data as shown in Figure 4, especially in terms of oxygen saturation (the parameter that was used for further correlation analysis).

### 3.2. Time-Varying Hemoglobin Concentration Profiles of DFU Subjects with Differing Skin Colors

When using optical imaging modalities (such as NIRS) that measure tissue oxygenation from the skin’s surface, light is significantly attenuated with an increase in melanin concentration in the epidermis (across skin colors). It is essential to account for this melanin-related attenuation (in the epidermis) to evaluate the changes in tissue oxygenation in the dermis and lower layers of the skin. In the current study, the tissue oxygenation changes are dynamically measured in response to a breath-hold stimulus, which is not expected to change the melanin concentration, but the underlying tissue oxygenation. Furthermore, the observed oxygenated flow changes in controls indicated that breath-holding may be suitable as a stimulus to assess the perfusion to wounds.

Spatial variations in skin pigmentation are more obvious in wounds as they heal. Herein, a feasibility study was performed on two DFU subjects in a UM-IRB (University of Miami-Internal review board) approved study at the University of Miami Wound Care Center [31]. This feasibility study was performed to assess if (i) breath-holding could induce oxygenated flow changes in DFUs apart from control subjects, and (ii) if variations in skin color impacts the dynamically changing tissue oxygenation measurements in response to the breath-hold stimulus. The recruited DFU subjects demonstrated a range of skin color (corresponding with Fitzpatrick grades of 1 to 5). Fitzpatrick skin type was confirmed by referencing the imaged tissue against a printed Fitzpatrick color scale. The first recruited DFU subject (subject 1 in Table 1) was a healed DFU case (as clinically assessed). The second subject (subject #2 in Table 1) was a post transmetatarsal amputation case imaged twice across weeks. During the two timepoints of imaging, the wound was clinically assessed as healing and non-healing. From a prior amputation, subject 2 had two varying skin colors around the DFU. The details of the recruited DFU subjects are listed in Table 1.

**Figure 5:**
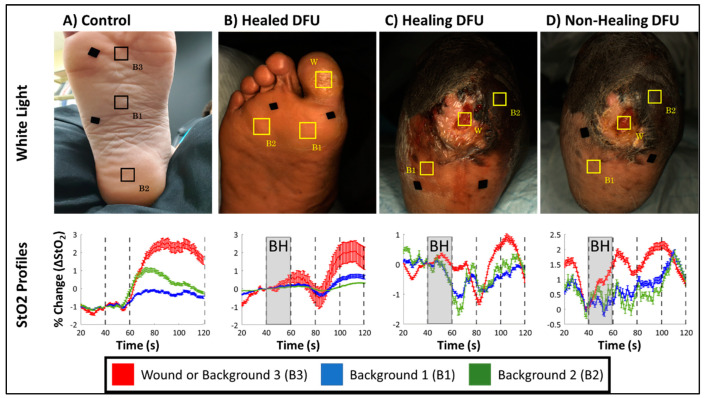
White light images for (**A**) a control case, (**B**) a healed DFU from subject-1, (**C**) a healing DFU (week 1) from subject-2, (**D**) and a non-healing DFU (week 6) case from subject-2, along with hemoglobin concentration profiles for ROIs selected over the wound (W) and background (B1, B2, and B3) tissue regions.

Both DFU subjects were imaged using the same breath-hold paradigm as that in the controls. The tissue oxygenation changes in response to breath-hold were compared across the DFU cases and in comparison to a control case in Figure 5. A 50 × 50-pixel ROI was selected over the (former) wound (W) and two background (B) tissue regions. For the second DFU subject, one ROI was selected in the proximal and distal end of the mid-foot to assess tissue oxygenation changes from lighter (B1) and darker (B2) skin regions (not necrotic), respectively. For the control case with no wounds, three ROIs were selected across the entire foot (B1, B2, and B3). The hemoglobin concentration profiles (in terms of ∆StO_2_) in response to the breath-hold is shown for all imaged cases and the selected ROIs. The pairwise Pearson’s correlation coefficient was calculated between ROIs within each case and provided in Table 2.

All DFU cases demonstrated an increase in ∆StO_2_ following breath-hold cessation like the sample control case (Figure 5), although, the increase in ∆StO_2_ was a delayed response in DFU cases (80–100 s) compared to the control case (60–80 s). This was consistently observed in all DFU cases (Figure 5B–D) and across the discrete wound and background locations demonstrating that DFU may have altered the underlying microcirculations spatially and potentially delayed the hemodynamic response accordingly. However, despite these differences in how ∆StO_2_ changes in response to the BH paradigm differ between control and DFU cases, the Pearson’s-based correlation was high between ∆StO_2_ signals acquired from background tissue regions (i.e., B1 vs. B2, B2 vs. B3 and B1 vs. B3) across all cases (Table 2).

Regardless of healing and disease status of the imaged tissue, all cases demonstrated strong correlation between their respective background regions (>84%). Furthermore, in healing and non-healing DFU cases from subject 2, the lighter (Fitzpatrick grade 2) and darker (Fitzpatrick grade 5) backgrounds ROIs were strongly correlated (>84%). This demonstrates that oxygenation flow patterns are synchronous, independent of the skin colors in the background regions. With regard to oxygenation changes in the wound, of the healing and non-healing DFU cases from subject 2, the wound was only weak to moderately correlated (26–57%) to the background region.

Hemoglobin concentration profiles provided insight into the oxygenation changes at a given tissue location but did not provide in-depth insight into the oxygenated flow changes across a 2D region of tissue. In the presence of a wound, the breath-hold response may be altered due to complications or the wound undergoing healing. To assess the synchrony of breath-hold induced oxygenation changes across the foot, correlation maps were calculated, as described in Section 2.4., for the cases in Figure 5.

### 3.3. Flow Correlation Maps in Controls Vs. DFUs

**Figure 6:**
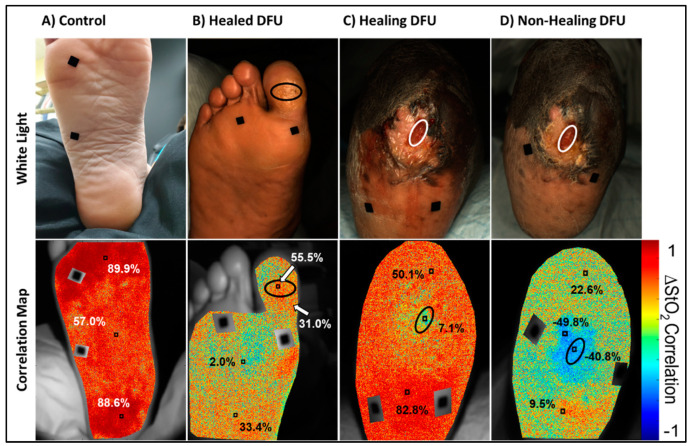
Oxygen saturation-based oxygenated flow correlation maps and color images for a (**A**) control, (**B**) healed DFU, and (**C**) healing DFU, and (**D**) a non-healing DFU case. Red in the correlation maps are regions that are positively correlated (+1), or synchronous, oxygenated flow changes with respect to the reference signal. Blue are regions that are negatively correlated (−1), or asynchronous, oxygenated flow changes with respect to the reference signal. The black ovals in the DFU maps denote the (former) DFU location, and the black squares are segmented out fiducial markers used for spatial referencing.

Oxygen saturation-based correlation maps were calculated for all control cases and the three DFU cases (across the two DFU subjects) are given in Figure 6. Flow correlation maps were generated using only the oxygen saturation-based hemoglobin parameter. Oxygen saturation was chosen because various past studies have suggested it as a potentially good indicator of wound healing [12,32]. In DFU subjects, flow patterns may differ between uninjured and wounded tissue regions. For the DFU subjects, the correlation maps were generated using a ROI-based reference signal selected from a region far away from the ulceration (i.e., the background) as the reference signal in Equation (1). A ROI-based reference signal can be used to select an uninjured region as a baseline for comparing the flow synchrony. From each correlation map, several sub-ROIs were selected. The average correlation coefficient was calculated for each sub-ROI to evaluate the flow synchrony at various points of the foot.

It was observed from Figure 6 that the control case was positively correlated across the entire sole of the foot. In addition, the whole foot had a similar degree of correlation (i.e., highly positively correlated across the foot) with respect to the average ∆StO_2_-based reference signal. Visually, this could be inferred from the primarily red coloration of the correlation map. The healed and healing DFU were both positively correlated across the foot, similar to the control case, but to a lesser degree. In the healed DFU, the distal and proximal sub-ROIs of the foot was more positively correlated than the middle sub-ROI (2%). In the healing DFU, the proximal (heel direction) sub-ROI was more correlated than the distal end. The wound region was also weakly correlated (7%) as compared to the background tissue.

The non-healing DFU case, however, had visually apparent negatively correlated (asynchronous) oxygenated flow in and around the DFU. The non-healing DFU correlation map had a region of negatively correlated tissue (correlation value of −40.8 to −49.8%) in and around the wound region, indicating asynchronous oxygenated flow and an overall low synchrony in the oxygenated flow response. Overall, the extent of (a)synchrony in the oxygenated flow may differ between healing and non-healing DFUs. While assessing the PCC at distinct regions can provide insight into how synchronous the oxygenated flow changes were, they do not adequately describe the overall flow synchrony across the foot.

The overall synchrony of the oxygenated flow across the imaged tissues was determined using the oxygenation flow index (OFI) as described in Section 2.4. Table 3 lists the average and standard deviation of the OFIs, as described in Section 2.4, calculated for each control subject across repetitions. OFIs were further calculated for the dorsum and sole of the foot to identify any potential differences in overall flow synchrony. All control subjects had positively correlated flow (or synchronous flow) in both the dorsum (~42%) and sole (~64%) of the foot. In controls, the sole side of the foot had higher OFI values than the dorsum side.

The OFIs of the healed and healing DFU cases are given in Table 4. The DFU subjects performed the breath-hold paradigm only once during their weekly clinical visit (unlike the 3 repetitions possible in control subjects). It was observed that the OFIs in healing and healed DFU case was 33% or greater (similar to that observed in controls in Table 3), unlike the non-healing DFU case which was closer to 0%. While the flow correlation maps and OFI assessment across the different cases is a feasibility study, an extensive correlation analysis across multiple DFU cases was part of another DFU imaging study [33]. In that study on 17 DFU cases, flow correlation-based threshold was developed to differentiate healing vs. non-healing DFUs from our spatio-temporal tissue oxygenation maps in response to the breath-hold paradigm [33]. A preliminary threshold (OFI < 28%) differentiated non-healing and complicated DFUs (infected or cases that had underlying issues or poor long term healing outcome) from healing DFUs. The overall oxygenation flow pattern was less synchronous (or the OFI value reduced) in the non-wound areas of the feet that were non-healing. In other words, the reduced OFI value (<28%) in the entire foot excluding the wound region is a possible indicator that the wound may not heal [33].

## 4. Discussion

### 4.1. Effect of Breath-Holding on Peripheral Oxygenation in Controls

Breath holding invokes cerebrovascular and cardiovascular changes to maintain oxygen supply to the brain and core functions. Due to this, breath-hold paradigms have been extensively used in two fields: (1) as a stimulus to produce cerebral vascular changes [19,34,35,36], and (2) to study the physiology mechanisms of the breath-holding. In this study, breath-holding was applied to assess its clinical applicability to produce peripheral tissue oxygenation changes. Overall, breath-holding is a complex physiological phenomenon invoking many biological mechanisms to conserve oxygen for critical functions. Aside from physiological conditions (lung volume, fitness, etc.), it is also mediated by the subjects resting state (exercising/stationary) and the environment itself (dry/wet).

Research into the cardiovascular side of the response often involves assessing heart rate, oxygen and carbon dioxide gas concentrations in the lungs and blood, arterial oxygen saturation, and blood pressure [22,37,38,39]. However, of relevance to this study has been the assessment of breath-hold induced tissue oxygenation changes in the peripheries. It is known that breath-holding induces peripheral vasoconstriction in the skin and skeletal muscles [37] which can reduce blood flow to the skin by as much as 40% [23]. However, the study of hemoglobin-based changes in peripheral tissues in response to breath-hold is limited [24,30,40].

Two studies were identified that imaged subjects under similar conditions to our current study. Both studies were conducted on subjects that were stationary and not immersed in water (unlike certain studies on divers immersed in water) to measure oxy- and deoxyhemoglobin [30] and oxygen saturation-based [24,30] changes on the left leg of recruited subjects. While these studies are not specifically focused on skin tissue oxygenation, other studies observed that skin and muscle oxygenation are affected by breath-hold induced peripheral vasoconstriction and cardiovascular regulation [22]. Grunovas et al. [30] and Bouten et al. [24] both reported a change in oxygenation parameters within the first 20 s of breath-holding. Oxyhemoglobin and oxygen saturation were reported to have begun decreasing 10 s after breath-hold onset, whereas deoxyhemoglobin was reported to have started increasing 5 s after breath-hold onset [24]. It was further reported that an overshoot of oxygen saturation was measured after breath-hold cessation [30].

In our current pilot study, a slight decrease in the oxygen saturation and a slight increase in deoxyhemoglobin was observed in the sole of the foot of control subjects during the breath-holding phase (shown in Figure 4F,H), although the increase started even before the breath-holding phase. Changes in oxyhemoglobin and total hemoglobin were not as distinct during the breath-hold phase in the sole of the foot. On the contrary, the extent of drop in deoxyhemoglobin and increase in oxygen saturation were distinct during the post breath-hold (or recovery) phase. Additionally, the greatest signal change was observed on the sole of the foot after breath-holding in comparison to changes observed in the dorsum of the foot.

Overall, the changes in tissue oxygenation parameters were greater during the recovery phase (or hyperemic phase) than during the breath-holding phase in both the dorsum and sole of the foot (as shown in Figure 4). An overshoot in oxygen saturation was observed during the recovery phase, as observed by other researchers as well [30]. An overshoot in oxyhemoglobin was also observed, along with an undershoot in deoxyhemoglobin. The overshoot in ∆HbO and ∆StO_2_ and an undershoot in ∆HbR continued until the end of the 60 s recovery phase. The total hemoglobin also increased during the 60 s recovery phase but to a lesser extent, as it is a summation of the increased oxy- and decreased deoxy-hemoglobin signal.

One potential influence that may affect the magnitude of the tissue oxygenation changes observed during the recovery phase is how subjects resumed normal breathing. Most subjects took a deep breath during the imaging studies after being informed to resume normal breathing. Deep inspiration induces a vasoconstrictive effect in the peripheries as measured from the fingers and is mediated by the sympathetic nervous system [41]—like breath-holding. One group published a summary of the relationship between respiration and peripheral vasoconstriction in their efforts to model the interactions [42]. They summarized that the degree of inspiration-based vasoconstriction is not as sensitive to the duration of the inspiration, but rather the change in lung volumes. Therefore, it stands to reason that the change in lung volume between residual volume and total lung capacity would illicit a strong vasoconstrictive response.

Around the 100th second time point in the paradigm, a change in tissue oxygenation trend was observed on the sole of the foot for all hemoglobin parameters (as seen in Figure 4). Oxyhemoglobin and oxygen saturation concentrations were observed to plateau and stabilize at a concentration ~2% greater than at breath-hold onset. The same was observed with total hemoglobin, but at a lower concentration (~0.5% greater). Inversely, deoxyhemoglobin was observed to reach its minimum value (~2.5% less) from baseline around the same 100th second, followed by a slight increase thereafter (as observed from Figure 4F). The observed change in trend might indicate a return to a new baseline, given the short duration of the breath-hold phase. While trends on both sides of the foot were similar during the recovery phase from 61 to 100 s, the overall extent of tissue oxygenation concentration changes on the dorsum was lesser than the sole. A possible reason could be due to superficial structures located on the dorsum, such as tendons and vasculature structures with varied flow patterns (further described in the paragraphs below). Overall, the strong hemoglobin-based response post breath-hold may indicate that, for short duration breath-holds, the greatest tissue oxygenation changes may be caused by the body attempting to reach a new baseline post stimulus.

In summary, regardless of potential variations between and within subjects, a 20 s breath-hold and a 60 s recovery phase demonstrated a consistent trend in all tissue oxygenation parameters across all subjects and repetitions in their imaged dorsum and sole of the foot. Independent of slight variations in how each subject performed the breath-holding and relaxation, the physiological changes in these tissue oxygenation parameters were similar. This demonstrates that the underlying phenomenon of peripheral vasoconstriction is consistent in all subjects and even upon repeated measurements. Hence, breath-hold based paradigms can serve as a potential stimulus to induce vasoconstriction in the peripheries to alter blood flow and hence, assess the adequacy of tissue oxygenation below the skin. The 80 s paradigm (20 s breath-hold and 60 s recovery phase) can be further shortened to observe the same changes in response to peripheral vasoconstriction when we apply a 40 s paradigm (20 s breath-hold and 20 s recovery phase). The increase in ∆HbO and ∆StO_2_ and the decrease in ∆HbR was distinctly observed even within the first 20 s of the recovery phase (as seen in Figure 4). Hence, the overall breath-hold paradigm was shortened to 40 s for further oxygenation flow correlation analysis.

### 4.2. Hemoglobin Concentration Profiles of DFUs with Varying Skin Colors

From the hemoglobin concentration profiles of the DFU subjects, it was observed that oxygen saturation increased post breath-holding-like controls. It was also noted that the response to breath-holding was more varied between the DFU cases. The variation in signals between DFUs could potentially reflect differences in how the breath-hold paradigm was performed by these subjects and possibly the differences in the imaged tissue location (post amputee vs. intact foot). In addition, the variations may be due to the extent of microcirculatory dysfunction between subjects at different stages in their treatment. DFU subjects, and diabetics as a whole, have been documented to have impaired microcirculation [43,44] that can be observed as early as the pre-diabetic phase [45]. The variation in breath-holding response may, in turn, reflect the underlying microcirculatory dysfunction. The microcirculatory impairment in diabetics may explain the asynchronous oxygenated flow response observed in the correlation maps of the non-healing DFU case. Quantification of the differences in oxygenated flow response between healing and non-healing DFUs is the subject of ongoing work.

From DFU subject 2, it was also observed that the signal trend between the open DFU and background regions differed. Melanin acts as an optical absorber that increases in concentration with darker skin colors. Hence, the signal intensity of the hemoglobin-concentrations profile plots was inherently influenced by the presence of melanin. In subject 2, the darker (Grade 5) and lighter (Grade 2) ROIs were more than 88% correlated in the healing and non-healing case. This indicated that the variation in hemoglobin signal was due to variations in flow changes and not from the contribution of melanin. While the background regions were strongly correlated in the healing and non-healing case, the wound region was only moderately correlated, between 26 and 57% (Table 2), to the background region for either case acquired from DFU subject 2. The moderate correlation between the wound and background region in the healing and non-healing case may reflect differing underlying causes, further supporting that in a temporal setting, the contribution of melanin to the measured hemoglobin-based signal may be neglectable. Temporal oxygenation monitoring allowed for an intensity-independent imaging approach across different skin colors. Our future efforts include enrolling a larger cohort of control subjects with different skin colors (possibly from different racial/ethnic groups) to systematically determine how an increase in melanin would affect the extent of change in tissue oxygenation in response to breath-hold.

### 4.3. Oxygenated Flow Response Differences Between Controls and DFUs

Controls: From the oxygenation flow correlation analysis across all control subjects, the calculated OFI from correlation maps of the sole and dorsum of the foot were positive (i.e., positively correlated). The positive tendency indicates that the oxygenated flow changes across the foot in response to breath-holding is overall synchronous (or similar). This was visually depicted in the control subject correlation map given in Figure 6. The OFI of the dorsum side correlation maps may partly be due to the superficial venous structures. Veins on the dorsum of the foot rank among the most superficial veins in the body [46]. These superficial, blood-filled structures may inherently have a different oxygenated flow response when compared to the surrounding tissue that diffusively receives their oxygen from capillaries. Regardless, the overall positive OFI values calculated on either side support a synchronous oxygenated flow response to breath-holding stimulus in controls.

DFUs: The flow correlation maps between the control case and DFU subjects differed. Unlike the healed and healing DFU case, the correlation map of the non-healing DFU region indicated an asynchronous oxygenated flow (with negative correlation values) in the wound bed and its immediate surroundings. The asynchrony in and around the wound region may indicate a compromise in the oxygenated flow to the foot, thus hindering healing. Additionally, the calculated OFI for the non-healing DFU was distinctly lower than the healed and healing DFU. The lower OFI demonstrates that there is an overall reduction in the oxygenation flow to the entire imaged foot region. Hence, the OFI could be a potential indicator of adequacy of oxygenation flows in terms of flow patterns (or synchrony) to assess if the DFUs are moving towards healing or still remain non-healing. The potential of breath-holding as a technique to demarcate altered oxygenated flow in DFUs using OFI is an ongoing study.

## 5. Conclusions

The objective of this study was to demonstrate the potential of breath-holding as a stimulus to induce peripheral tissue oxygenation changes. Our non-invasive, non-contact NIROS device was used to image breath-hold induced tissue oxygenation changes in the feet of control subjects. It was observed from controls that the trend in hemoglobin-based oxygenation changes in response to breath-hold was consistent across repeated measurements and across all subjects. A 40 s breath-hold paradigm with 20 s breath-hold and 20 s post breath-hold (or recovery phase) was sufficient to induce peripheral vasoconstriction and related tissue oxygenation changes in the feet of all subjects. In addition, breath-hold induced oxygen saturation changes were synchronous across the feet of control subjects, demonstrating that the oxygenation flow or perfusion is similar or uniform.

In parallel, a case study was conducted on two DFU subjects to determine if breath-holding induced tissue oxygenation changes were comparable to controls. In was observed that regardless of healing status, there was an increase in oxygen saturation after breath-hold. It was further observed that oxygenated flow changes acquired from differing skin color in the same subject were strongly correlated (>84%). In conclusion, breath-hold paradigms have potential as a stimulus to assess the oxygenated flow patterns, overcoming the effects of melanin, towards assessing DFU healing status.

Our future imaging studies will involve extensive clinical studies on control subjects across all Fitzpatrick skin colors (1–6) to systematically evaluate the effectiveness of our proposed breath-holding paradigm, followed by studies on DFUs. The imaging studies on DFUs will employ the breath-hold stimulus-based diffuse reflectance and tissue oxygenation measurements to determine the flow correlation maps in DFUs longitudinally across weeks of healing and across all Fitzpatrick skin colors. This systematic study will better characterize the oxygenation flow index as a biomarker to determine the potential for a wound to heal or not, independent of the skin color.

## 6. Patents

A Non-Provisional US Patent has been filed related to the work reported in this manuscript.

## Figures and Tables

**Table 1:** Details of the two DFUs cases assessed for the effect of skin tone.

Case	DFU Subject	Status	Side	Week of Treatment	DFU Location	Fitzpatrick Grade
**1**	1	Healed	Right	4	Big Toe (Plantar)	1
**2**	2	Healing	Left	1	Midfoot (Post Transmetatarsal amputation)	2 (Proximal) 5 (Distal)
**3**	2	Non-Healing	Left	6	Midfoot (Post Transmetatarsal amputation)	2 (Proximal) 5 (Distal)

**Table 2:** Pearson’s correlation coefficients calculated between ROIs for one control case and the 3 DFU cases. * Indicates cases with low correlation coefficients between wound and backgrounds.

DFU Subject	Region	Pearson Correlation Coefficient
**Control** **(Subject 1)**	B1 vs. B2	95.42%
	B1 vs. B3	95.2%
	B2 vs. B3	85.5%
**1** **(Healed DFU)**	B1 vs. B2	93.9%
	W vs. B1	95.5%
	W vs. B2	84.0%
**2** **(Week 1—Healing DFU)**	B1 vs. B2	92.8%
	W vs. B1 *	26.1%
	W vs. B2 *	31.5%
**2** **(Week 6—Non-Healing DFU)**	B1 vs. B2	84.8%
	W vs. B1 *	57.2%
	W vs. B2 *	34.3%

**Table 3:** Average of the oxygenation flow index (OFI) by subject and imaged foot side.

Controls	Dorsum OFI	Sole OFI
1	36.6 ± 5.5%	60.2 ± 25.0%
2	45.0 ± 12.1%	59.6 ± 20.5%
3	45.0 ± 19.7%	72.2 ± 22.0%
Grand Average	42.2 ± 12.6%	64.0 ± 20.5%

**Table 4:** Oxygenation flow index (OFI) in DFU cases.

DFUs	OFI
Healed	33.4%
Healing	68.4%
Non-healing	9.5%

## Data Availability

The datasets presented in this article are not readily available because of privacy and ethical reasons involving data from subjects with diabetic foot ulcers from a wound care center. Requests to access the datasets should be directed to the corresponding author at godavart@fiu.edu.
